# Conditional targeting in mice reveals that hepatic homogentisate 1,2-dioxygenase activity is essential in reducing circulating homogentisic acid and for effective therapy in the genetic disease alkaptonuria

**DOI:** 10.1093/hmg/ddz234

**Published:** 2019-10-10

**Authors:** Juliette H Hughes, Ke Liu, Antonius Plagge, Peter J M Wilson, Hazel Sutherland, Brendan P Norman, Andrew T Hughes, Craig M Keenan, Anna M Milan, Takao Sakai, Lakshminarayan R Ranganath, James A Gallagher, George Bou-Gharios

**Affiliations:** 1 Institute of Ageing and Chronic disease, University of Liverpool, Liverpool, L7 8TX, UK; 2 Institute of Translational Medicine, University of Liverpool, Liverpool, L69 3GA, UK; 3 Liverpool Clinical Laboratories, Department of Clinical Biochemistry and Metabolic Medicine, Royal Liverpool and Broadgreen University Hospitals Trust, Liverpool, L7 8XP, UK

## Abstract

Alkaptonuria is an inherited disease caused by homogentisate 1,2-dioxygenase (HGD) deficiency. Circulating homogentisic acid (HGA) is elevated and deposits in connective tissues as ochronotic pigment. In this study, we aimed to define developmental and adult HGD tissue expression and determine the location and amount of gene activity required to lower circulating HGA and rescue the alkaptonuria phenotype.

We generated an alkaptonuria mouse model using a knockout-first design for the disruption of the HGD gene. Hgd tm1a −/− mice showed elevated HGA and ochronosis in adulthood. LacZ staining driven by the endogenous HGD promoter was localised to only liver parenchymal cells and kidney proximal tubules in adulthood, commencing at E12.5 and E15.5 respectively. Following removal of the gene trap cassette to obtain a normal mouse with a floxed 6^th^ HGD exon, a double transgenic was then created with Mx1-Cre which conditionally deleted HGD in liver in a dose dependent manner. 20% of HGD mRNA remaining in liver did not rescue the disease, suggesting that we need more than 20% of liver HGD to correct the disease in gene therapy.

Kidney HGD activity which remained intact reduced urinary HGA, most likely by increased absorption, but did not reduce plasma HGA nor did it prevent ochronosis. In addition, downstream metabolites of exogenous ^13^C_6_-HGA, were detected in heterozygous plasma, revealing that hepatocytes take up and metabolise HGA.

This novel alkaptonuria mouse model demonstrated the importance of targeting liver for therapeutic intervention, supported by our observation that hepatocytes take up and metabolise HGA.

## Introduction

Alkaptonuria (AKU; OMIM #203500) is a rare metabolic recessive disease where the enzyme homogentisate 1,2-dioxygenase (HGD; EC 1.13.11.5), which is mainly found in the liver, is deficient (1). Garrod in 1908 unveiled the term *inborn error of metabolism* and proposed that AKU was caused by the lack of an enzyme that in normal individuals split the aromatic ring of homogentisic acid (HGA) (2). Biochemical evidence of the defect in AKU was provided by La Du in 1958, where he demonstrated the absence of HGD activity in a liver homogenate prepared from an AKU patient and established that the failure to synthesize active enzyme was the sole cause of AKU (1).

HGD deficiency leads to HGA accumulation in the blood and tissues, despite urinary excretion. It has been proposed that excess HGA undergoes oxidation and polymerization to form a dark brown ochronotic pigment (3) that deposits in connective tissues such as the skin, sclera, spine and articular cartilage, as well as in heart valves (4,5), where it causes aortic stenosis (6). AKU patients suffer from early-onset severe osteoarthropathy based on premature degeneration of articular cartilage, and disease manifestations worsen with age. Despite liver deficiency of HGD, the main pathophysiological manifestation of AKU relates to the function of non-metabolized HGA in the joints.

HGD has been mapped onto human chromosome 3q13.33 (http://www.ncbi.nlm.nih.gov/gene/3081) (7,8). Currently, 203 different HGD pathogenic variants have been identified (HGD mutation database: http://hgddatabase.cvtisr.sk) (9,10), of which the most frequent are missense variants (representing 68.3%), followed by splicing (13.4%) and frameshift (11.3%) mutations (11). In addition to the liver, HGD is thought to be expressed in the prostate, small intestine, colon and kidney (12), as well as in osteoarticular compartment cells (chondrocytes, synoviocytes and osteoblasts) (13), and the brain (14). However, this has not been verified by *in situ* hybridisation or HGD labelling.

Currently, AKU treatment is palliative via analgesics and joint replacement, with dietary protein restriction and vitamin C showing little or no efficacy (15). Recently, there have been trials of 2-(2-nitro-4-trifluoromethylbenzoyl)-1,3-cyclohexanedione (NTBC), more commonly referred to as nitisinone, in the treatment of AKU (16). Nitisinone, which inhibits the HGA-producing enzyme 4-hydroxyphenylpyruvate dioxygenase (HPPD; EC 1.13.11.27), has been the licensed treatment for hereditary tyrosinaemia type 1 (HT-1; OMIM #276700) since 2002, where fumarylacetoacetate hydroxylase (FAH; EC 3.7.1.2) deficiency results in liver failure, hepatocellular carcinoma and renal tubular dysfunction (17). Moreover, the off-licence use of nitisinone at the National Alkaptonuria Centre (Liverpool, UK) has shown a decreased rate of disease progression in addition to lowering serum and urine HGA (18).

Treatment of other inborn errors of metabolism related to the phenylalanine/tyrosine pathway by enzyme replacement has been attempted with some success (19). In the past decade, liver-directed gene therapy has emerged as a promising alternative to transplantation in monogenic liver disorders such as AKU (20). The level of HGD required to rescue the disease, if expression outside the liver can affect the phenotype and whether circulating HGA can be metabolised by HGD-expressing cells are essential questions that must be addressed before such treatments are investigated.

To answer these questions, a new targeted knockout-first AKU mouse model was generated. This mouse harbours a LacZ reporter gene within the HGD locus for localizing gene expression and was conditionally manipulated to obtain an inducible and liver-specific knockout. This study provides compelling evidence that targeting hepatic HGD plays an indispensable role in any enzyme replacement or gene therapy for AKU.

## Results

### Generation of the conditionally targeted Hgd mouse

**Figure 1:**
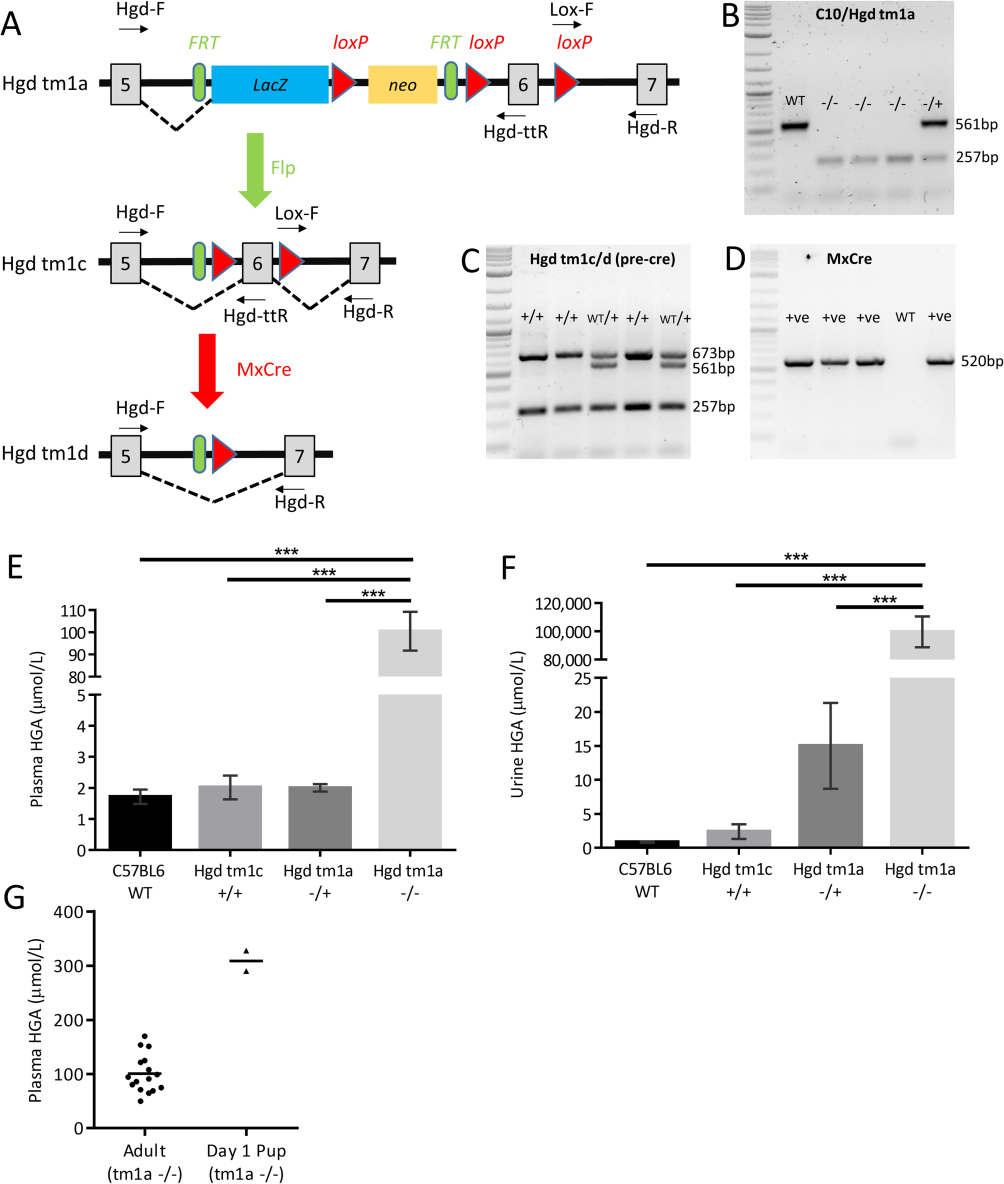
Phenotyping of Hgd tm1a mice. **A**–**D** shows genotyping of the modified HGD allele. A schematic of the modified HGD allele is shown in A. Hgd tm1a: AKU phenotype. Hgd tm1c: wild-type phenotype. Hgd tm1d: liver-specific and inducible KO. Using primer pairs Hgd-F/Hgd-ttR and Lox-F/Hgd-R, B shows the genotyping of tm1a; C shows the genotyping of tm1c (after flp recombination) and tm1d (before cre recombination). D shows the genotyping of MxCre. A 2-log DNA ladder was used in B–D. **E**–**H** show elevation of HGA in Hgd tm1a −/−. E and F show plasma and urine HGA. Plasma HGA is elevated approximately 100-fold in Hgd tm1a −/− mice (*n* = 16) in contrast to C57BL/6 wild-type (*n* = 4), Hgd tm1c +/+ (*n* = 7) and Hgd tm1a −/+ (*n* = 18) controls. Urinary HGA is elevated approximately 10 000-100 000-fold in Hgd tm1a −/− mice (*n* = 19) in contrast with C57BL/6 wild-type (*n* = 7), Hgd tm1c +/+ (*n* = 7) and Hgd tm1a −/+ (*n* = 19) controls. **G** shows HGA levels in Day 1 Hgd tm1a −/− pups (two pools of *n* = 3) in contrast with adults (*n* = 16). HGA = homogentisic acid. Significance: *P* < 0.05^*^, *P* < 0.01^**^ and *P* < 0.001^***^. Error bars represent SEM.

ES cells from clone C10 resulted in chimeras which achieved germline transmission. This knockout-first allele (Hgd tm1a) contained an IRES:LacZ gene trap cassette and a promoter-driven neo cassette inserted into the fifth HGD intron with the sixth exon flanked by loxP sequences (see Fig. 1A) (21, 22). Homozygous Hgd tm1a mice showed an AKU phenotype based on HGD gene disruption. In C10/tm1a mice, Lox-F/Hgd-R primers amplified the loxP sequence (257 bp) showing the allele was floxed (Fig. 1). Hgd-F/Hgd-ttR primers produced a 561 bp band in the wild-type allele; the gene trap cassette sequence in the modified allele was too large to be amplified. Homozygous tm1a therefore had only the 257 bp floxed band, heterozygotes had both the floxed 257 bp and wild-type 561 bp bands and wild-type had only the 561 bp band (Fig. 1B).

Hgd tm1c +/+ had restored HGD gene expression based on removal of the gene trap and was phenotypically wild-type. To achieve inducible and conditional HGD deletion, interferon inducible Mx-1 was used to drive expression of cre recombinase, MxCre (23), generating the Hgd tm1d allele. In the post-flp tm1c and tm1d (before cre recombination), Lox-F/Hgd-R primers showed that the allele was floxed (257 bp) and Hgd-F/Hgd-ttR primers showed that Flp recombination had occurred (673 bp). Wild-type tm1c/d had only the 561 bp band (Hgd-F/Hgd-ttR). Homozygous tm1c/d (+/+) had both the 257 bp floxed and 673 bp post-flp bands, and heterozygous tm1c/d (WT/+) had all three bands (257 bp floxed, 673 bp post-flp and 561 bp wild-type) (see Fig. 1C). Primers produced a band at 520 bp when MxCre was present (Fig. 1D).

### Elevation of HGA in plasma and urine

HGA was measured in urine and plasma of mice (Fig. 1E and F). Urinary HGA (mean ± SEM) was elevated approximately 100 000-fold (99 575 ± 30 851 μmol/L) and plasma HGA elevated about 100-fold (100.5 ± 34.9 μmol/L) in the Hgd tm1a −/− mice in contrast to Hgd tm1a −/+ (plasma: 2.0 ± 0.5 μmol/L; urine: 15 ± 25.3 μmol/L), Hgd tm1c +/+ (plasma: 2.0 ± 1.0 μmol/L; urine: 2.4 ± 2.9 μmol/L) and C57BL/6 wild-type (plasma: 1.7 ± 0.6 μmol/L; urine: 0.8 ± 0.2 μmol/L) mice. These differences were all statistically significant (*P* < 0.001; one-way ANOVA, Tukey–Kramer post hoc).

As AKU is present at birth, plasma HGA was measured in Day 1 Hgd tm1a −/− pups (two pools of *n* = 3; gestation: 19.5 days) (see Fig. 1G). Plasma HGA (mean ± SEM) was elevated 3-fold in Day 1 pups (308.8 ± 18.9 μmol/L) in contrast with adult Hgd tm1a −/− mice (100.5 ± 8.7 μmol/L).

### Detection of ochronosis and its progression

**Figure 2:**
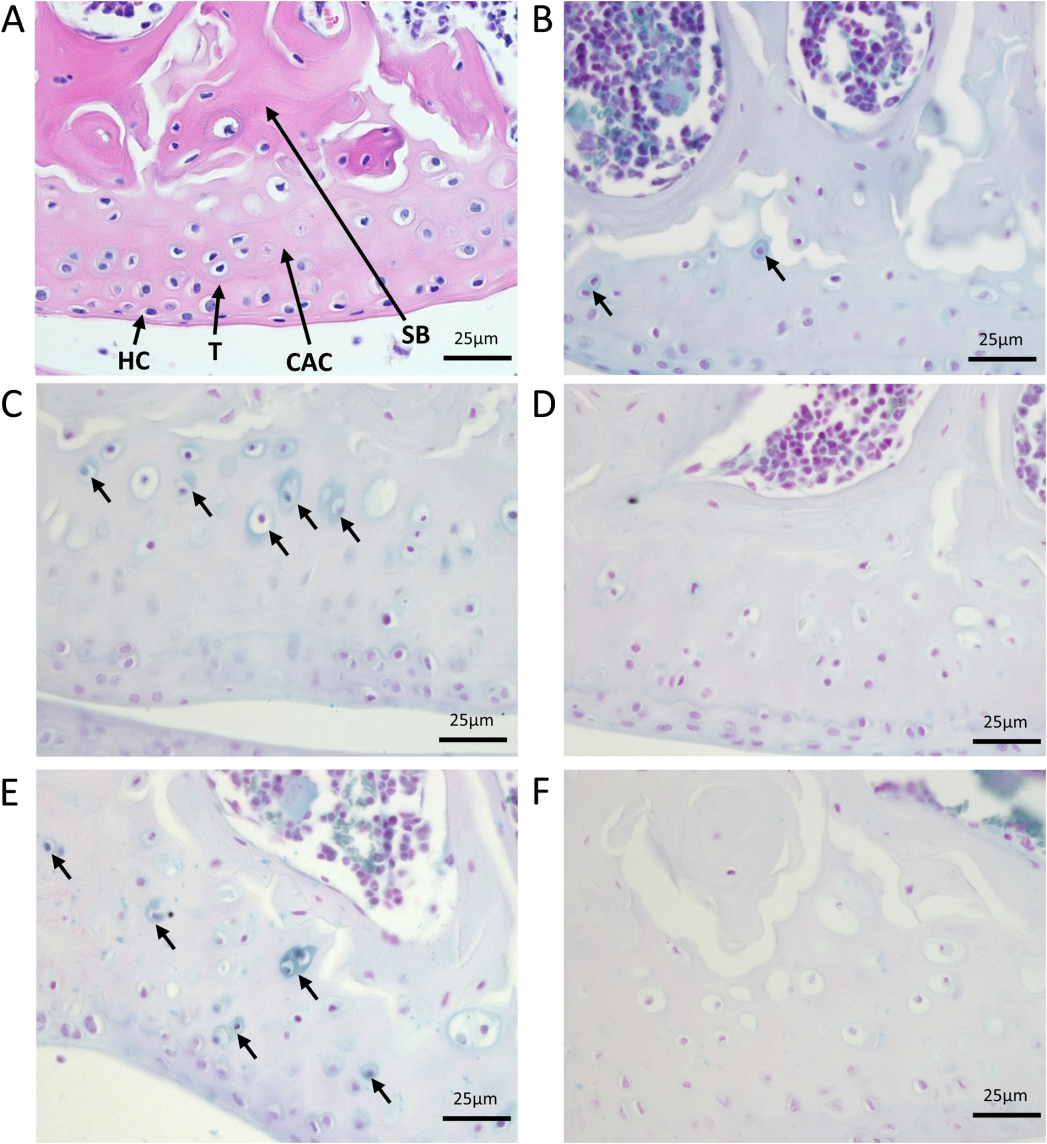
Initiation and progression of ochronosis in Hgd tm1a mice. H&E staining in **A** shows division of articular cartilage into different zones: hyaline cartilage (HC) and calcified articular cartilage (CAC) separated by the tidemark (T). Deep to calcified articular cartilage is subchondral bone (SB). **B**–**F** show femoral condyles from Hgd tm1a mice that have been Schmorl’s-stained (stains ochronotic pigment a blue colour). B shows pericellular pigmentation of chondrons situated in the calcified articular cartilage in a 9-week Hgd tm1a −/− femoral condyle. C and E show the femoral condyle of Hgd tm1a −/− mice at 26 and 40 weeks, respectively. The pigmentation has advanced to the inner compartment of the cell, with more numerous affected chondrocytes showing varying pigment intensities. The pigmentation is still confined to the calcified articular cartilage layer. Hgd tm1a −/+ mice at 26 and 40 weeks (D and F respectively) show no pigmentation. All sections: 6 μm.

Knee joints from Hgd tm1a −/− mice aged 7–40 weeks were examined for pigmentation. Ochronosis was found in calcified articular cartilage (Fig. 2A), first appearing at 9 weeks (Fig. 2B). The pigment was initially pericellular (9–11 weeks) and very infrequent. At 26 and 40 weeks (Fig. 2C and E, respectively), the number and intensity of pigmented chondrons were increased and showed advancement to the intracellular compartment. Clusters of pigmented chondrons were seen at ligament attachment sites (not shown). At 40 weeks, pigmentation was still confined to calcified cartilage. Heterozygous controls showed no pigmentation at 26 and 40 weeks (Fig. 2D and F, respectively).

### Adult HGD expression

**Figure 3:**
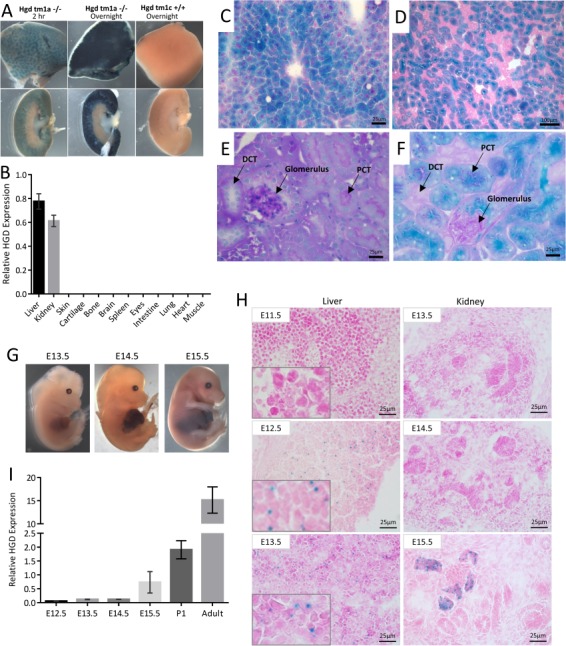
Localization of HGD expression. **A** shows LacZ staining in adult liver (top row) and kidney (bottom row). LacZ staining (blue) represents HGD expression seen only in the liver parenchyma and kidney cortex of Hgd tm1a −/− adult mice. Control Hgd tm1c +/+ liver and kidney were LacZ-negative. **B** shows HGD mRNA across a variety of adult Hgd tm1d WT tissues analysed via qPCR (HGD1 primers). **C**–**F** shows frozen section staining of adult liver and kidney from Hgd tm1a −/− mice, with liver (C) and kidney cortex (D) showing specific LacZ staining after 2 h. In E, PAS staining distinguishes PCTs from DCTs by the presence of a PAS-positive dark pink/purple brush border. In F, the LacZ blue cells localize with the PAS-positive PCT cells. **G**–**I** shows HGD expression in Hgd tm1a −/− embryos. G shows whole time-mated embryo LacZ staining, with positive staining seen at E14.5 in the liver. H shows microscopic frozen section LacZ staining of liver and kidney. Positive staining is seen at E12.5 onwards in liver and from E15.5 in kidney. I shows liver HGD mRNA (HGD1 primers) in E12.5 (*n* = 3), E13.5 (*n* = 3), E14.5 (*n* = 4) and E15.5 (*n* = 3) embryos, Day 1 pups (P1) (*n* = 6) and adult Hgd tm1a −/− mice (*n* = 4). All sections: 6/7 μm. PAS: periodic acid–Schiff. PCT = proximal convoluted tubule. DCT = distal convoluted tubule. Error bars represent SEM. HGD expression normalised to 18S.

Adult tissues from Hgd tm1a −/− were stained for LacZ to visualize HGD expression as blue staining. Positive staining was present in the liver and kidney cortex after 2 h which intensified when left overnight (Fig. 3A). Heterozygous staining was less intense (not shown) and was slower to develop. Hgd tm1c wild-type-like liver and kidney did not stain (Fig. 3A). All other Hgd tm1a tissues investigated including the brain, heart, lung, muscle, spleen, intestine, skin, bone, cartilage, eye and prostate were negative. HGD mRNA analysis via qPCR (HGD1 primers spanning Exons 3–4 before gene trap cassette) confirmed this staining pattern, with HGD expression only present in the liver and kidney (Fig. 3B).

LacZ in the whole liver and kidney was limited by the penetration of substrate; therefore, frozen section staining was undertaken to demonstrate that LacZ and therefore HGD is expressed in the cytoplasm throughout the liver parenchyma (Fig. 3C). In the kidney cortex, glomeruli were LacZ-negative, and only certain tubules stained positive (Fig. 3D), which were identified using periodic acid–Schiff for brush border staining in proximal convoluted tubules (PCTs) (Fig. 3E and F).

### Embryonic HGD expression

To determine when HGD is expressed, LacZ staining of time-mated Hgd tm1a −/− embryos was carried out. Whole embryo staining showed positive LacZ staining in the liver at E14.5 and onwards (Fig. 3G). However, histological sections revealed punctate staining at E12.5 and onwards in the liver (Fig. 3H). Positive LacZ staining was seen in the kidney at E15.5 in some of the developing kidney tubules (Fig. 3H). All other embryonic tissues examined in frozen sections, including brain, eye, bones and other internal organs, were LacZ-negative.

Similarly, liver HGD mRNA expression in Hgd tm1a −/− (HGD1 primers spanning Exons 3–4 before gene trap cassette) was analysed by qPCR (Fig. 3I) at E12.5 (*n* = 3), E13.5 (*n* = 3), E14.5 (*n* = 4) and E15.5 (*n* = 3), Day 1 pups (*n* = 6) and in contrast with adult mice (*n* = 4, male, mean age 19.7 weeks). In contrast to the adult, liver HGD expression was 0.3% at E12.5, 0.8% at both E13.5 and E14.5 and 2.3% at E15.5. This considerable difference in HGD mRNA was reflected in the LacZ staining intensity seen between the adult liver (Fig. 3C) in contrast to the embryo liver (Fig. 3H). Day 1 pup liver HGD expression was 12.6% of the adult expression level (8-fold lower).

### Inducible and liver-specific HGD knockout

**Figure 4:**
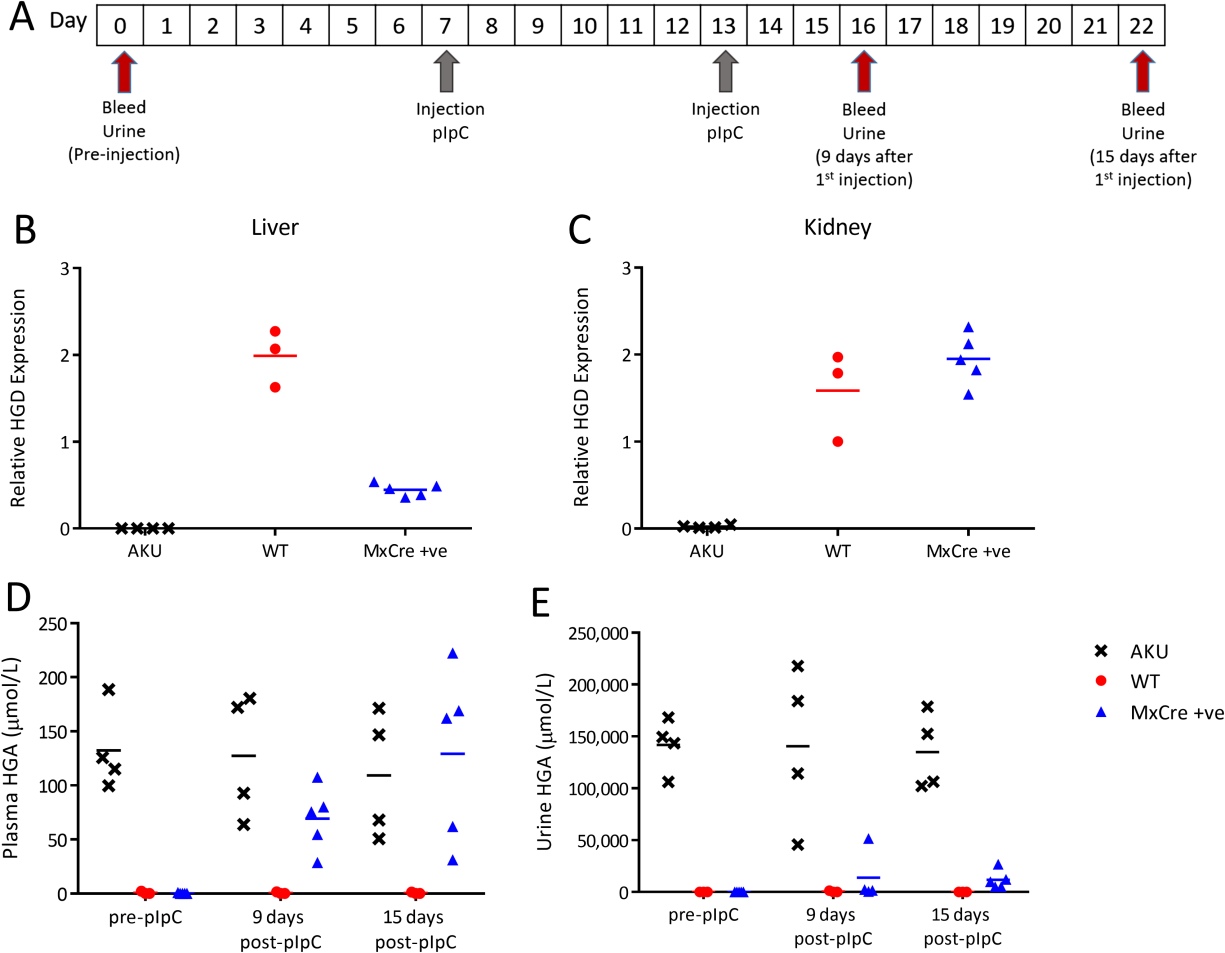
Liver-specific HGD knockout, induced by pIpC injection, in Hgd tm1d MxCre +ve mice. **A** shows the timescale of blood and urine sampling and pIpC injections. **B** and **C** show HGD mRNA (HGD2 primers; relative to 18S) in Hgd tm1a −/− (AKU controls, *n* = 4), Hgd tm1d MxCre WT (wild-type controls, *n* = 3) and Hgd tm1d MxCre +ve mice (MxCre +ve, *n* = 5) in the liver and kidney, respectively, 15 days after pIpC injection. MxCre +ve mice show reduced liver, but not kidney, HGD expression in contrast to wild-type controls. **D** and **E** show plasma and urine HGA, respectively, pre-injection, 9 and 15 days after pIpC injection. Pre-injection, HGA in the plasma is not detected in MxCre +ve or WT mice. Hgd tm1a −/− mice show high HGA. Post-pIpC, plasma HGA in MxCre +ve mice is increased. Pre-injection, urinary HGA is low in MxCre +ve and WT mice in contrast to Hgd tm1a −/−. Post-pIpC, urinary HGA shows a relatively small increase in MxCre +ve mice.

To investigate the effect of liver-specific HGD gene deletion, double transgenic Hgd tm1d MxCre +ve mice (*n* = 5) and wild-type (*n* = 3) and AKU (*n* = 4) controls were injected with pIpC. Blood and urine samples were collected according to the scheme in Figure 4A. Fifteen days after the first pIpC injection, MxCre +ve mice showed a 77.6% decrease in liver HGD mRNA in contrast with wild-type controls (Fig. 4B). Kidney HGD expression in MxCre +ve mice did not change and was comparable to wild-type controls (Fig. 4C). AKU mice had no HGD mRNA expression as expected (primers span Exons 9–10 after gene trap cassette).

Following the knockout of approximately 78% liver HGD mRNA in MxCre +ve mice, mean plasma HGA (± SEM) increased from 0.2 ± 0.2 to 129.3 ± 35.6 μmol/L at 15 days post-pIpC (Fig. 4D). MxCre +ve urinary HGA (Fig. 4E) was elevated from 9.4 ± 7.1 to 11 807 ± 974 μmol/L at 15 days post-pIpC but remained low in comparison to AKU controls (134 948 ± 18 479 μmol/L). Both plasma and urinary HGA remained high in AKU controls and low in wild-type controls as expected.

### Long-term liver-specific HGD knockout

**Figure 5:**
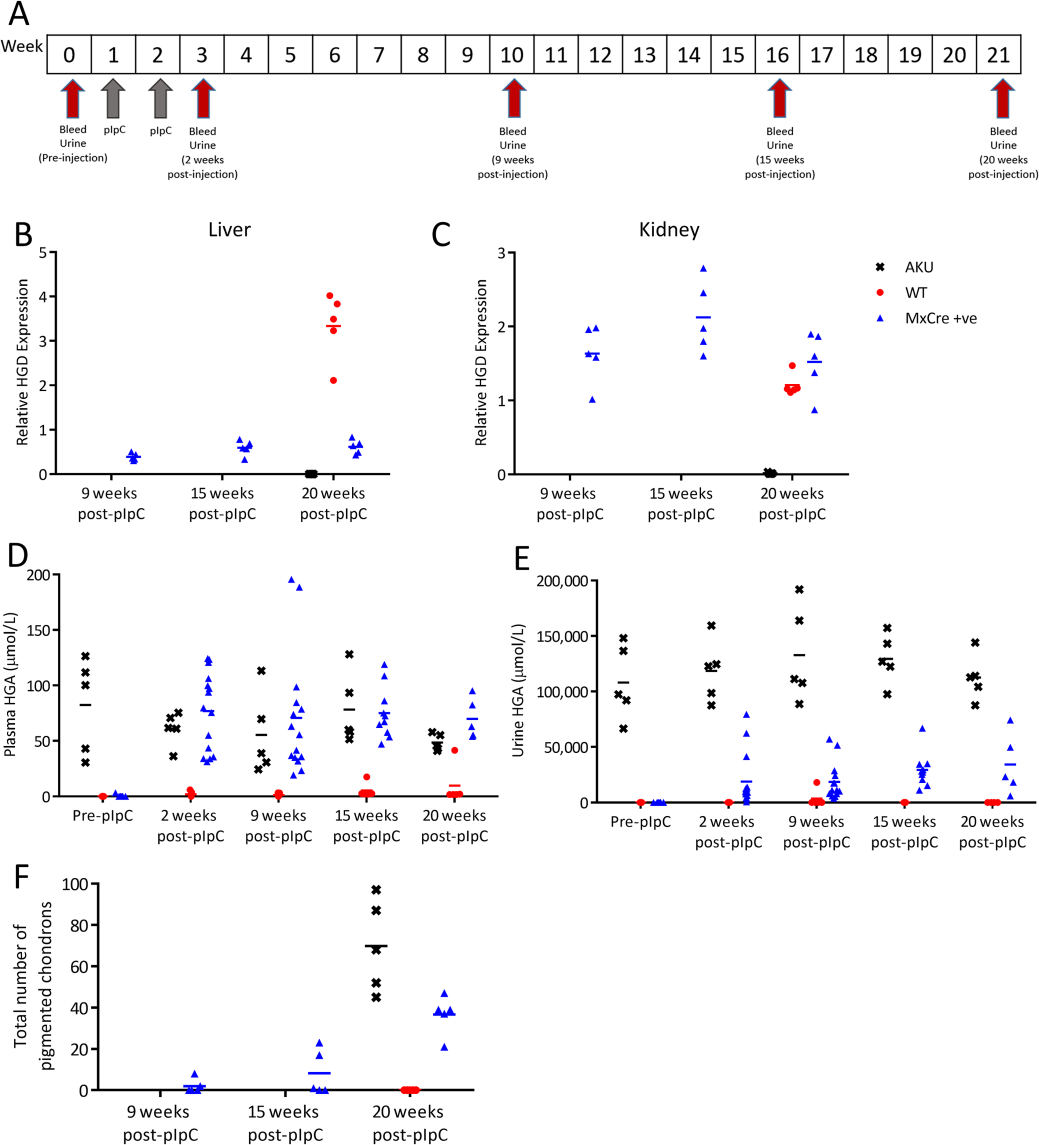
Long-term follow-up of Hgd tm1d MxCre +ve mice injected with pIpC. **A** shows the timescale of blood and urine sampling and pIpC injections. Hgd tm1a −/− were injected with pIpC (AKU controls, *n* = 5). Hgd tm1d MxCre +ve were injected with PBS (wild-type controls, *n* = 5). Hgd tm1d MxCre +ve mice were injected with pIpC (MxCre +ve, *n* = 15). **B** and **C** show relative HGD mRNA (HGD2 primers; relative to 18S) in the liver and kidney, respectively, of MxCre +ve mice at 9, 15 and 20 weeks after the first injection, and in AKU and wild-type controls at 20 weeks. Liver HGD mRNA was reduced, in contrast to wild-type controls, in MxCre +ve mice at 9 weeks and was sustained until 20 weeks. Kidney expression was not reduced by pIpC injection in MxCre +ve mice. **D** and **E** show plasma and urine HGA levels, respectively, pre-injection and at 2, 9, 15 and 20 weeks post-injection. Pre-injection, plasma HGA is not detected in MxCre +ve or WT mice and AKU mice showed elevated HGA. Post-pIpC, plasma HGA in MxCre +ve mice was increased at 2 weeks and remained at this level until 20 weeks. Pre-injection, urinary HGA is low in MxCre +ve and WT mice in contrast with AKU controls. Post-pIpC, urinary HGA showed a relatively small increase in MxCre +ve mice in contrast with AKU controls. The total number of pigmented chondrons in a representative section of the knee joint, stained with Schmorl’s, is shown in F, at 9, 15 and 20 weeks post-injection.

MxCre +ve mice (*n* = 15) and AKU controls (*n* = 5) were injected with pIpC. MxCre +ve mice were injected with PBS (*n* = 5) as mice. Injections and sampling of blood and urine were carried out according to the scheme in Figure 5A. MxCre +ve mice injected with pIpC were culled at 9 (*n* = 5), 15 (*n* = 5) and 20 (*n* = 5) weeks post-injection, with wild-type and AKU controls culled at 20 weeks. Liver and kidney mRNA was taken, and knee joints were taken to assess ochronosis.

As with the previous study, liver HGD mRNA was reduced in pIpC-injected MxCre +ve mice, which was sustained to 20 weeks post-injection (see Fig. 5B). In contrast to wild-type controls, liver HGD expression was 11.7, 17.7 and 18.4% at 9, 15 and 20 weeks, respectively. Kidney HGD expression in the MxCre +ve mice remained comparable to wild-type controls (Fig. 5C). The reduction of liver HGD mRNA in the MxCre +ve mice subsequently caused plasma HGA to increase (Fig. 5D) to a level comparable with AKU controls. Urinary HGA (Fig. 5E) was increased in the MxCre +ve mice but not to that of AKU controls. Knee sections stained with Schmorls’ stain were scored to obtain the number of pigmented chondrons found in a representative knee joint section (Fig. 5F). Few or no pigmented chondrons were found in the MxCre +ve knee joints 9 weeks post-pIpC, increasing in number at 15 and 20 weeks. Wild-type controls showed no pigmentation.

### Liver-specific HGD knockout: dose response

**Figure 6:**
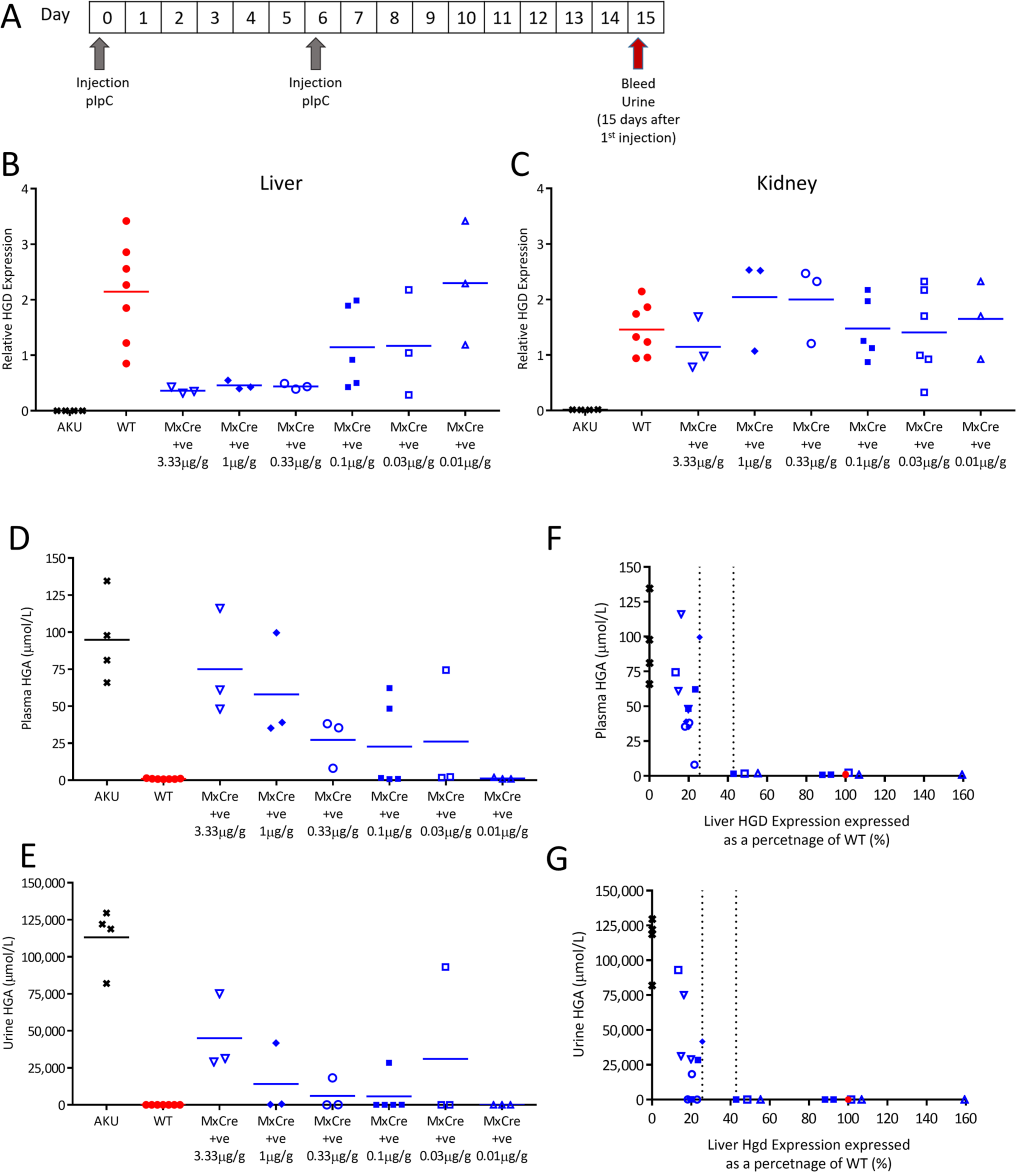
Liver-specific HGD knockout, induced by decreasing doses of pIpC, in Hgd tm1d MxCre +ve mice. **A** shows the timescale of blood and urine sampling and injections of pIpC. **B** and **C** show relative HGD expression (HGD2 primers; relative to 18S) in Hgd tm1a −/− (AKU controls, *n* = 4), Hgd tm1d MxCre WT (wild-type controls, *n* = 7) and Hgd tm1d MxCre +ve mice (3.33 μg/g, *n* = 3; 0.1 μg/g, *n* = 3; 0.33 μg/g, *n* = 3; 0.1 μg/g, *n* = 5; 0.03 μg/g, *n* = 3; 0.01 μg/g, *n* = 3) in the liver and kidney respectively, 15 days post-pIpC. Varying the dose of pIpC resulted in a range of liver HGD expression in the MxCre +ve mice, with lower doses resulting in higher HGD expression. Kidney HGD expression was unchanged by pIpC. **D** and **E** show plasma and urine HGA, respectively, 15 days post-pIpC. A dose response in HGA was observed in both the plasma and the urine of MxCre +ve mice, with higher pIpC doses resulting in more elevated HGA. HGA was elevated in Hgd tm1a −/− and was low in wild-type controls. **F** and **G** show the relationship between liver HGD mRNA (expressed as a percentage of the mean wild-type level) with plasma and urinary HGA respectively, 15 days post-pIpC.

In order to investigate the effect of varying liver HGD mRNA expression levels on the AKU phenotype, a short-term dose response study was carried out. Figure 6A demonstrates the study design. MxCre +ve mice were given two injections of pIpC at the following doses: 3.33 μg/g (*n* = 3), 1 μg/g (*n* = 3), 0.33 μg/g (*n* = 3), 0.1 μg/g (*n* = 5), 0.03 μg/g (*n* = 3) and 0.01 μg/g (*n* = 3) body weight. AKU (Hgd tm1a −/−; *n* = 4) and wild-type (MxCre WT; *n* = 7) controls were given the highest dose of 3.33 μg/g pIpC. With the exception of the 0.01 μg/g group, liver HGD mRNA in MxCre +ve mice was reduced at 15 days post-injection (Fig. 6B) in all groups, with a dose response observed at the lower pIpC doses. The mean liver HGD expression in MxCre +ve mice 15 days post-pIpC in contrast to wild-type controls was lowered to 17.0, 20.5, 20.5, 53.4 and 54.5% with decreasing pIpC doses from 3.33 to 0.03 μg/g. The mean liver HGD expression in the 0.01 μg/g group was comparable to the wide-type controls. Kidney HGD expression was unchanged (Fig. 6C).

Plasma HGA in MxCre +ve mice at 15 days post-pIpC were lowered in a dose-responsive manner (mean ± SEM) to 75.0 ± 20.9, 57.9 ± 20.9, 27.2 ± 9.6, 22.7 ± 13.4, 26.1 ± 24.1 and 1.3 ± 0.4 μmol/L with decreasing pIpC doses (Fig. 6D). Similarly, urine HGA demonstrated a dose response at 15 days post-pIpC, at (mean ± SEM) 45 074 ± 14 977, 14 169 ± 13 783, 6137 ± 6064, 5717 ± 5667, 31 043 ± 31 041 and 2.0 ± 0.2 μmol/L with decreasing pIpC doses (Fig. 6E). These levels of urine HGA were elevated in contrast with wild-type (1.9 ± 0.2 μmol/L) controls, but lower than AKU (113 067 ± 10 609 μmol/L) controls. Figure 6F and G show the relationship between liver HGD mRNA (expressed as percentage of the mean wild-type level) and HGA.

### Metabolism of circulating HGA

**Figure 7:**
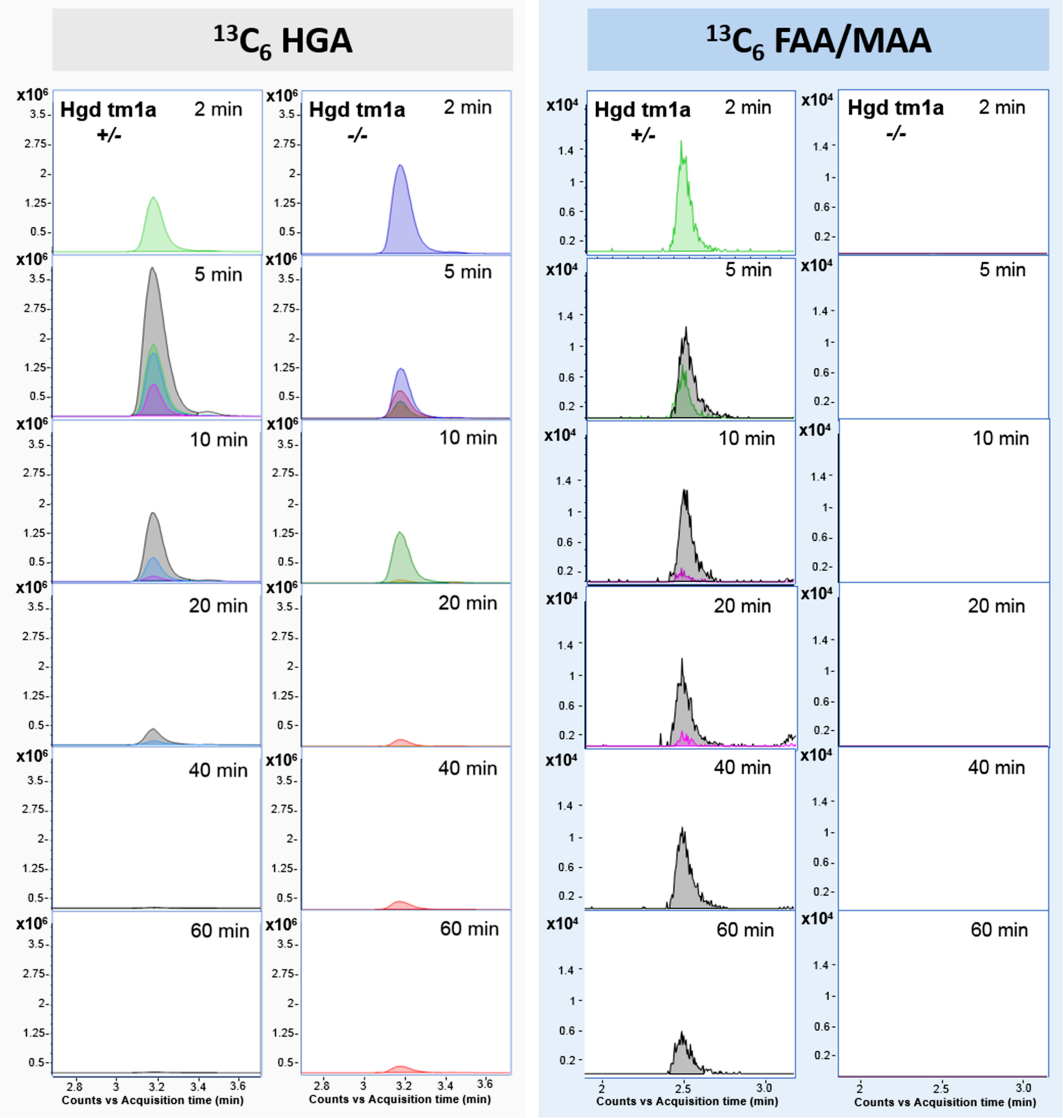
LC-QTOF-MS plasma flux analysis data acquired following injection of Hgd tm1a −/− and Hgd tm1a −/+ mice with ^13^C_6_-HGA. Plots show extracted ion chromatograms (theoretical accurate mass ± 5 ppm) for ^13^C_6_-HGA and ^13^C_6_-fumarylacetoacetic acid (FAA)/^13^C_6_-maleylacetoacetic acid (MAA). Traces are for individual plasma samples taken 2–60 min post-injection and colour-coded to represent individual mice. ^13^C_6_-HGA was detected in Hgd tm1a −/− and Hgd tm1a −/+ indicating presence of the tracer. ^13^C_6_-MAA/FAA was observed at 2–60 min post-injection in Hgd tm1a −/+ and was not observed in Hgd tm1a −/− at any time point.

To determine if circulating HGA can be taken up by HGD-expressing cells to be metabolized intracellularly, both Hgd tm1a −/− (*n* = 4) and Hgd tm1a −/+ (*n* = 4) mice were injected with ^13^C_6_-HGA into the tail vein. Plasma samples were then collected at various time points, from 2–60 min post-injection. HGA in its native form (m + 0) was detected in plasma of Hgd tm1a −/− mice at all time points and was absent in Hgd tm1a −/+ mice (not shown). ^13^C_6_-HGA was detected in both Hgd tm1a −/− and −/+ mice after injection (Fig. 7). M + 6 isotopologues of fumarylacetoacetic acid (^13^C_6_-FAA)/maleylacetoacetic acid (^13^C_6_-MAA), ^13^C_6_-labelled downstream metabolites, were detected in Hgd tm1a −/+ mice after ^13^C_6_-HGA injection and were not detected in Hgd tm1a −/− mice. Native FAA/MAA was not detected in either Hgd tm1a −/− or −/+ mice (not shown).

## Discussion

Management of inborn errors of metabolism such as AKU has traditionally consisted of diet and supportive therapy. However, other treatment options have become available, including enzyme inhibition (17,24), enzyme replacement (19), cell and organ transplantation (20), gene therapy (20) and CRISPR technology (25).

The pathophysiology of AKU has been investigated in *ex vivo* tissue samples (14,26,27) and *in vitro* models (13,28), but to investigate the metabolic consequences of AKU and novel therapeutic approaches, we have generated a well-characterized animal model. This model will be used to investigate all aspects of AKU pathophysiology including the mechanism of ochronosis and any associated tissue changes including amyloidosis (29). We have generated a HGD knockout-first mouse model that included a beta galactosidase (LacZ) gene trap within the HGD gene locus, which has enabled precise localization of HGD expression. Targeted gene disruption in Hgd tm1a removes any potentially confounding mutations that could be present in an existing ENU (N-ethyl-N-nitrosourea) AKU mouse model (30), as ENU mutagenesis causes a high frequency of genomic mutations (31). This new mouse recapitulated the human disease. Manipulation of this Hgd tm1a knockout-first allele by FRT/flp and Cre/loxP recombination enabled liver-specific HGD deletion in double-transgenic Hgd tm1d MxCre +ve mice, highlighting important considerations for future therapy in AKU.

Plasma HGA in Hgd tm1a −/− is comparable to that previously reported values in the ENU AKU mouse (30). This mutagenesis model exhibited the first signs of ochronosis at 15 weeks (30). Knee joints were therefore examined from 7 to 11 weeks in Hgd tm1a −/− mice, with pericellular pigmentation identified at 9 weeks; progression was then similar to mutagenesis AKU mice. Ochronosis in the mouse appears to represent the early stages of human joint pathophysiology in AKU with pigmentation confined to individual chondrocytes and their territorial matrix in the calcified cartilage.

The LacZ reporter gene has enabled both temporal and spatial histological localisation of HGD showing that HGD was expressed throughout the liver parenchyma and kidney PCT cells. It has previously been suggested that HGD is expressed in the intestine and prostate (12), brain (14) and bone/cartilage (13), but this was not evident using this knock-in LacZ reporter gene, nor by qPCR analysis of HGD mRNA.

LacZ staining of time-mated embryos demonstrated that hepatic HGD expression begins at E12.5 (Fig. 3H), confirmed with qPCR analysis of liver HGD mRNA from E12.5 to E15.5. Hepatic cords, containing hepatoblasts, have formed by E10.0 in the developing liver alongside haematopoietic cells. The liver then expands as a result of hepatoblast proliferation and haematopoietic activity from E10.5 to E11.5 (32). Haematopoietic activity rapidly increases, peaking at E13.5, and does not start to decline until E15.5 (32). Haematopoietic cells encompass almost 75% of total liver volume at E13.0 (33) with hepatoblasts at E13.5 having limited contact with each other (32), explaining the diffuse and punctate LacZ staining and low HGD mRNA level in the embryonic liver. Murine hepatoblasts begin to differentiate into hepatocytes at E14.5 (32). Haematopoietic activity continues into the first post-natal week, which may explain why Day 1 pups have about an eighth of adult HGD expression (32). In the kidney, adult LacZ staining suggests that the LacZ-positive cells seen at E15.5 are developing PCT cells of the nephron.

HGD expression begins in embryonic development. The 3-fold greater HGA level seen at birth in contrast to adulthood in Hgd tm1a −/− mice (Fig. 1G) highlights the importance of HGD, even at this very early time point, perhaps suggesting that therapeutics strategies, such as nitisinone (16) or gene/enzyme replacement, should ideally begin in early life or at birth.

### Liver-specific HGD deletion

To investigate the contribution of non-hepatic HGD towards HGA metabolism, double transgenic mice were generated by mating floxed Hgd tm1d mice with an MxCre recombinase line and used for liver-specific HGD deletion (23, 34). Two doses of 10 μg/g body weight pIpC (Figs 4 and 5) resulted in an approximate 80% reduction in liver HGD mRNA, whilst kidney HGD mRNA was maintained at the wild-type level. This reduction of liver HGD mRNA and subsequent increase in plasma HGA to a level comparable with AKU controls suggests that hepatic HGD is crucial for HGA metabolism.

These results indicate that future gene/enzyme replacement therapy should target the liver to combat elevated plasma HGA that causes ochronosis in AKU. This is supported by a case report of an AKU patient receiving a liver transplant, after which they found no HGA in the urine and reported a halt in progressive arthropathy (35). Full liver HGD mRNA knockout was not achieved with two 10 μg/g doses in the present study. Further reducing liver HGD mRNA would not provide further insight into the level of liver HGD mRNA required to rescue the phenotype.

The data here reveals that approximately 20% liver HGD mRNA, delivered by gene therapy for example, will not rescue AKU; elevated HGA subsequently caused ochronosis in the knee joints of the mice (Fig. 5F). Determining how much liver HGD is required to rescue the phenotype is an important question for future therapy. The dose response study here (Fig. 6) intended to estimate how much liver HGD mRNA would significantly lower circulating HGA. A dose response was observed in both the plasma and urine HGA levels (Fig. 6D and E). However, for liver HGD mRNA, there was only a dose response between 0.33 and 0.01 μg/g pIpC. One possible explanation for HGD mRNA not corresponding to plasma HGA in the other dose groups could be that the mRNA:protein ratio is not linear. Thus, we suggest that the minimum level of liver HGD mRNA required to eliminate circulating HGA must fall between the dashed lines (Fig. 6F), between 26 and 43% of liver HGD mRNA.

The intact kidney HGD mRNA did not have an impact on plasma HGA, but instead caused reduced urinary HGA in contrast to AKU controls. In 2002, an AKU patient who received a kidney transplant reportedly had normalized plasma HGA and decreased urinary HGA (both approximately half pre-transplant levels) (36). Liver-specific HGD deletion shown here in mice however suggests that kidney HGD mRNA is unlikely to rescue the AKU phenotype as blood HGA levels were elevated. The improvement reported with kidney transplantation was likely based on improved renal elimination as the patient had renal failure and subsequently very high HGA pre-transplant, rather than donor HGD expression. Indeed, in the conditional mouse model (Figs 4 and 5), we were expecting high HGA in the urine when circulating HGA increased. However, in the liver-specific knockout, the urine level did not increase, suggesting HGA reabsorption and subsequent metabolism by the intact kidney HGD, for which we do not know the mechanism. Fundamentally, the importance of this finding is that intact kidney HGD does not rescue the disease and therefore liver HGD is critical for the correction of AKU.

### HGD activity and HGA metabolism: considerations for gene therapy

Restoring HGD activity in all liver cells, via gene therapy or enzyme replacement, is unachievable by any current method, but the data here suggests that it is not necessary. In heterozygous mice and humans, one functioning copy of the HGD gene in all cells is sufficient to deal with HGA metabolism. Assuming heterozygous mice possess 50% of the HGD mRNA in contrast with wild-types (adult LacZ staining suggests this assumption is correct), then 50% HGD mRNA in every hepatocyte can rescue AKU. As described previously, conditional deletion to 20% of total HGD liver mRNA did not appear to rescue the phenotype. However, we cannot determine how many cells were expressing the gene, either as one or two alleles, nor the distribution of expression which could be variable from cell to cell. The proportion of corrected cells and the level of therapeutic gene expression per cell required to rescue AKU is therefore still not clear. A recent study providing the first human genotype–phenotype correlation data for the three most frequent HGD mutation variants (33 patients homozygous for these variants) identified in the SONIA-2 trial demonstrated no difference in baseline visit serum or 24 h urine HGA, or clinical symptoms such as eye pigmentation, hip bone density or degree of scoliosis between patients predicted to have 1% (16 patients) or 31–34% (17 patients) residual HGD catalytic activity as determined *in vitro* against wild-type recombinant human HGD enzymes (9).

Another consideration for gene therapy is that hepatocytes without sufficient HGD activity to metabolize HGA could lead to its accumulation in the bloodstream. In this study, intravenous injection of isotopically labelled HGA (Fig. 7) provides evidence that circulating HGA can re-enter HGD-expressing cells to be metabolized by intracellular HGD. Thus, it should not be necessary to repair 100% of liver cells because HGA produced by AKU hepatocytes could be taken up and metabolized by genetically repaired cells. This model represents a paradigm for inherited liver metabolic diseases, in particular, genetic disorders of tyrosine metabolism.

## Conclusion

In summary, this new targeted HGD knockout mouse exhibits the characteristic traits of AKU. Both adult and embryo HGD expression has been localized to only the liver and kidney using a reporter gene within the HGD locus. More importantly, the conditional Hgd tm1d MxCre model has highlighted the importance of liver HGD expression in limiting the pathological effects of the HGA pool despite apparent reabsorption of HGA in the kidney.

## Materials and Methods

### Generation of HGD knockout-first mice

Two ES cell lines (clones Hgd C10, Hgd C11) were obtained from the UC Davis KOMP repository. They were grown on feeder cells as described (37). Healthy ES colonies were injected into blastocysts of C57BL/6 (Harlan, UK) and chimeras born from both lines. Only C10 chimeras achieved germline transmission. All mice were housed and maintained within the University of Liverpool Biological Services Unit in specific pathogen-free conditions in accordance with UK Home Office guidelines. Food and water were available *ad libitum*.

### Urine and blood collection

Tyrosine pathway metabolites in acidified urine and plasma from venous tail bleeds were analysed via high-performance liquid chromatography (HPLC) tandem mass spectrometry assays (38,39).

### LacZ staining: tissues/embryos

Embryos from E13.5 to E16.5 were stained for ß-galactosidase as previously described (40). Adult tissues were stained via the same protocol with size-adjusted fixation times.

### LacZ staining: frozen sections

Frozen liver and kidney sections (6 μm) were fixed (0.2% glutaraldehyde in PBS) for 10 min. After three washes in cold PBS, they were stained for ß-galactosidase as mentioned previously and counterstained with eosin. Whole embryos were fixed as mentioned previously, transferred to 30% sucrose overnight and then embedded in OCT on dry ice. Frozen sections (7 μm) were LacZ-stained at room temperature overnight.

### qPCR

RNA was extracted using the Qiagen RNeasy Mini Kit, reverse transcription of RNA was carried out using the Applied Biosystems RNA-to-cDNA kit and qPCR was performed using the Bio-Rad iQ™ SYBR® Green Supermix. HGD1 primers are forward 5′-TGTCCACGGAACACCAATAA-3′ and reverse 5′-GCCAACTTCATCCCAGTTGT-3′. HGD2 primers are forward 5′-GACCCATCGGAGCAAATGGC-3′ and reverse 5′-AGTGTAACCACCTGGCACTC-3′. 18S (housekeeping gene) primer sequences are forward 5′-TGTCCACGGAACACCAATAA-3′ and reverse 5′-AGTTCTCCAGCCCTCTTGGT-3′. 18S was not affected by pIpC administration.

### Ochronosis

Coronal knee joint paraffin sections were Schmorl’s-stained and counterstained with nuclear fast red (28, 41) to identify pigmented chondrons. Scoring of all four joint quadrants was carried out blind to experimental conditions and genotype.

### Hgd tm1d conditional knockout

To obtain the conditional Hgd tm1d line, Hgd tm1a mice were crossed with Flpo mice to remove the FRT-flanked gene trap cassette, leaving a floxed target exon (Hgd tm1c) (42). Homozygote-floxed Hgd tm1c mice were crossed with MxCre mice. The removal of the floxed 6^th^ exon was induced with two intraperitoneal injections of polyinosinic:polycytidylic acid (pIpC) at 10 μg/g body weight (43). Hgd tm1a −/− mice were injected with pIpC as AKU controls, and wild-type controls were either Hgd tm1d MxCre WT injected with pIpC or Hgd tm1d MxCre +ve mice with PBS. HGA was measured in plasma and urine samples collected pre-injection and at time points post-injection. Liver and kidney HGD mRNA was analysed. In the long-term study, knee joints were collected for ochronosis scoring. For the dose response study, the same protocol was followed as mentioned previously, using diluted pIpC at doses 3.33 to 0.01 μg/g body weight.

### Isotopic HGA injection

Hgd tm1a −/− (*n* = 4) and Hgd tm1a −/+ (*n* = 4) mice were injected with ^13^C_6_-HGA into the lateral tail vein, adjusted to body weight to achieve a final blood concentration of ~1 mmol/L. Under anaesthesia, venous tail bleeds were collected at time points post-injection, ranging from 2 to 60 min. Whole blood was centrifuged, and the supernatant removed and immediately frozen.

Non-targeted metabolic flux analysis was performed to trace metabolism of ^13^C_6_-HGA. Metabolic profiling was performed using a published mass spectrometric technique (44). Briefly, plasma was diluted 1:9 plasma:deionized water and HPLC performed on an Atlantis dC_18_ column (3 × 100 mm, 3 μm, Waters, UK) coupled to an Agilent (Cheadle, UK) 6550 quadrupole time-of-flight mass spectrometer. An accurate-mass compound database with potential association to HGA was generated for data mining using Agilent Pathways to PCDL. Data were mined for these compound targets with an accurate mass window of ± 5 ppm using ‘batch isotopologue extraction’ in ProFinder (build 08:00, Agilent). Isotopologue extraction investigates association with the injected ^13^C_6_-HGA by examining the relative abundances of the M + 0–M + 6 isotopologues for compound targets.

### Statistical analysis

Statistical analysis was performed using Stats Direct 3 statistical software (UK). Significance is denoted as *P* < 0.05 ^*^, *P* < 0.01^**^ and *P* < 0.001^***^.

## Funding

Alkaptonuria Society (J.H.H.).

## Author contributions:

G.B.G., L.R.R. and J.A.G. designed the study. G.B.G., K.L., A.P. and J.H.H. established the mouse model. J.H.H., K.L., P.J.M.W., H.S., B.P.N. and C.M.K. carried out the laboratory analyses. A.T. and A.M. assisted in data acquisition. T.S. supplied Cre mice and knowledge of pIpC. L.R.R., J.A.G. and G.B.G. supervised the project. J.H.H. wrote the first draft of the paper. All authors reviewed the content and agreed the final version.

*Conflict of Interest statement*. The authors declare no competing interests.

## References

[1] La Du B.N., Zannoni V.G., Laster L. and Seegmiller J.E. (1958) The nature of the defect in tyrosine metabolism in alcaptonuria. J. Biol. Chem., 230, 250–261. 13502394

[2] Garrod A.E. (1902) The incidence of alkaptonuria: a study in chemical individuality. Lancet, 160, 1616–1620.

[3] O’Brien W.M., La Du B.N. and Bunim J.J. (1963) Biochemical, pathologic and clinical aspects of alcaptonuria, ochronosis and ochronotic arthropathy. Am. J. Med., 34, 813–838.

[4] Phornphutkul C., Introne W.J., Perry M.B., Bernardini I., Murphey M.D., Fitzpatrick D.L., Anderson P.D., Huizing M., Anikster Y., Gerber L.H. et al. (2002) Natural history of alkaptonuria. N. Engl. J. Med., 347, 2111–2121. 12501223 10.1056/NEJMoa021736

[5] Helliwell T.R., Gallagher J.A. and Ranganath L. (2008) Alkaptonuria - a review of surgical and autopsy pathology. Histopathology, 53, 503–512. 18336562 10.1111/j.1365-2559.2008.03000.x

[6] Hannoush H., Introne W.J., Chen M.Y., Lee S.J., O’Brien K., Suwannarat P., Kayser M.A., Gahl W.A. and Sachdev V. (2012) Aortic stenosis and vascular calcifications in alkaptonuria. Mol. Genet. Metab., 105, 198–202. 22100375 10.1016/j.ymgme.2011.10.017 PMC3276068

[7] Montagutelli X., Lalouette A., Coudé M., Kamoun P., Forest M. and Guénet J.L. (1994) AKU, a mutation of the mouse homologous to human alkaptonuria, maps to chromosome 16. Genomics, 19, 9–11. 8188247 10.1006/geno.1994.1004

[8] Manning K., Fernandez-Canon J.M., Montagutelli X. and Grompe M. (1999) Identification of the mutation in the alkaptonuria mouse model. Hum. Mutat., 13, 171–171. 10.1002/(SICI)1098-1004(1999)13:2<171::AID-HUMU15>3.0.CO;2-W 10094559

[9] Ascher D.B., Spiga O., Sekelska M., Pires D.E.V., Bernini A., Tiezzi M., Kralovicova J., Borovska I., Soltysova A., Olsson B. et al. (2019) Homogentisate 1,2-dioxygenase (HGD) gene variants, their analysis and genotype–phenotype correlations in the largest cohort of patients with AKU. Eur. J. Hum. Genet., 27, 888–902. 30737480 10.1038/s41431-019-0354-0 PMC6777518

[10] Zatkova A., Sedlackova T., Radvansky J., Polakova H., Nemethova M., Aquaron R., Dursun I., Usher J.L. and Kadasi L. (2012) Identification of 11 novel homogentisate 1,2 dioxygenase variants in alkaptonuria patients and establishment of a novel LOVD-based HGD mutation database. JIMD Rep., 4, 55–65. 23430897 10.1007/8904_2011_68 PMC3509877

[11] Zatkova A. (2011) An update on molecular genetics of Alkaptonuria (AKU). J. Inherit. Metab. Dis., 34, 1127–1136. 21720873 10.1007/s10545-011-9363-z

[12] Fernández-Cañón J., Granadino B., Beltrán-Valero de Bernabé D., Renedo M., Fernández-Ruiz E., Peñalva M. and Rodríguez de Córdoba S. (1996) The molecular basis of alkaptonuria. Nat. Genet., 14, 19–24. 8782815 10.1038/ng0996-19

[13] Laschi M., Tinti L., Braconi D., Millucci L., Ghezzi L., Amato L., Selvi E., Spreafico A., Bernardini G. and Santucci A. (2012) Homogentisate 1,2 dioxygenase is expressed in human osteoarticular cells: implications in alkaptonuria. J. Cell. Physiol., 227, 3254–3257. 22105303 10.1002/jcp.24018 PMC3427883

[14] Bernardini G., Laschi M., Geminiani M., Braconi D., Vannuccini E., Lupetti P., Manetti F., Millucci L. and Santucci A. (2015) Homogentisate 1,2 dioxygenase is expressed in brain: implications in alkaptonuria. J. Inherit. Metab. Dis., 38, 807–814. 25762405 10.1007/s10545-015-9829-5

[15] Ranganath L.R., Jarvis J.C., Gallagher J.A. and Ranganath L.R. (2013) Recent advances in management of alkaptonuria (invited review; best practice article). J Clin Pathol, 66, 367–373. 23486607 10.1136/jclinpath-2012-200877

[16] Ranganath L.R., Milan A.M., Hughes A.T., Dutton J.J., Fitzgerald R., Briggs M.C., Bygott H., Psarelli E.E., Cox T.F., Gallagher J.A. et al. (2014) Suitability of Nitisinone in Alkaptonuria 1 (SONIA 1): an international, multicentre, randomised, open-label, no-treatment controlled, parallel-group, dose-response study to investigate the effect of once daily nitisinone on 24-h urinary homogentisic acid. Ann. Rheum. Dis., 1, 1–6. 10.1136/annrheumdis-2014-206033 25475116

[17] Ashorn M., Pitkanen S., Salo M.K. and Heikinheimo M. (2006) Current strategies for the treatment of hereditary tyrosinemia type I. Pediatr. Drugs, 8, 47–54. 10.2165/00148581-200608010-00004 16494511

[18] Ranganath L.R., Khedr M., Milan A.M., Davison A.S., Hughes A.T., Usher J.L., Taylor S., Loftus N., Daroszewska A., West E. et al. (2018) Nitisinone arrests ochronosis and decreases rate of progression of alkaptonuria: evaluation of the effect of nitisinone in the United Kingdom National Alkaptonuria Centre. Mol. Genet. Metab., 125, 127–134. 30055994 10.1016/j.ymgme.2018.07.011

[19] Harding C.O. (2017) Gene and cell therapy for inborn errors of metabolism In Inherited Metabolic Diseases. Springer Berlin Heidelberg, Berlin, Heidelberg, pp. 155–171.

[20] Brunetti-Pierri N. (2008) Gene therapy for inborn errors of liver metabolism: progress towards clinical applications. Ital. J. Pediatr., 34, 2. 19490653 10.1186/1824-7288-34-2 PMC2603013

[21] Skarnes W.C., Rosen B., West A.P., Koutsourakis M., Bushell W., Iyer V., Mujica A.O., Thomas M., Harrow J., Cox T. et al. (2011) A conditional knockout resource for the genome-wide study of mouse gene function. Nature, 474, 337–342. 21677750 10.1038/nature10163 PMC3572410

[22] Collins F.S. and Rossant J. (2007) A mouse for all reasons. Cell, 128, 9–13. 17218247 10.1016/j.cell.2006.12.018

[23] Kühn R., Schwenk F., Aguet M. and Rajewsky K. (1995) Inducible gene targeting in mice. Science, 269, 1427–1429. 7660125 10.1126/science.7660125

[24] Milan A.M., Hughes A.T., Davison A.S., Devine J., Usher J., Curtis S., Khedr M., Gallagher J.A. and Ranganath L.R. (2017) The effect of nitisinone on homogentisic acid and tyrosine: a two-year survey of patients attending the National Alkaptonuria Centre. Liverpool. Ann. Clin. Biochem., 54, 323–330. 28081634 10.1177/0004563217691065

[25] Yin H., Xue W., Chen S., Bogorad R.L., Benedetti E., Grompe M., Koteliansky V., Sharp P.A., Jacks T. and Anderson D.G. (2014) Genome editing with Cas9 in adult mice corrects a disease mutation and phenotype. Nat. Biotechnol., 32, 551–553. 24681508 10.1038/nbt.2884 PMC4157757

[26] Taylor A.M., Wlodarski B., Prior I.A., Wilson P.J.M., Jarvis J.C., Ranganath L.R. and Gallagher J.A. (2010) Ultrastructural examination of tissue in a patient with alkaptonuric arthropathy reveals a distinct pattern of binding of ochronotic pigment. Rheumatology, 49, 1412–1414. 20181670 10.1093/rheumatology/keq027

[27] Taylor A.M., Boyde A., Wilson P.J., Jarvis J.C., Davidson J.S., Hunt J.A., Ranganath L.R. and Gallagher J.A. (2011) The role of calcified cartilage and subchondral bone in the initiation and progression of ochronotic arthropaththy in alkaptonuria. Arthritis Rheum., 63, 3887–3896. 22127706 10.1002/art.30606

[28] Tinti L., Taylor A.M., Santucci A., Wlodarski B., Wilson P.J., Jarvis J.C., Fraser W.D., Davidson J.S., Ranganath L.R. and Gallagher J.A. (2011) Development of an in vitro model to investigate joint ochronosis in alkaptonuria. Rheumatology, 50, 271–277. 20952450 10.1093/rheumatology/keq246

[29] Millucci L., Braconi D., Bernardini G., Lupetti P., Rovensky J., Ranganath L. and Santucci A. (2015) Amyloidosis in alkaptonuria. J. Inherit. Metab. Dis., 38, 797–805. 25868666 10.1007/s10545-015-9842-8

[30] Preston A.J., Keenan C.M., Sutherland H., Wilson P.J., Wlodarski B., Taylor A.M., Williams D.P., Ranganath L.R., Gallagher J.A. and Jarvis J.C. (2014) Ochronotic osteoarthropathy in a mouse model of alkaptonuria. and its inhibition by nitisinone. Ann. Rheum. Dis., 73, 284–289. 23511227 10.1136/annrheumdis-2012-202878

[31] Justice M.J., Noveroske J.K., Weber J.S., Zheng B. and Bradley A. (1999) Mouse ENU mutagenesis. Hum. Mol. Genet., 8, 1955–1963. 10469849 10.1093/hmg/8.10.1955

[32] Crawford L.W., Foley J.F. and Elmore S.A. (2010) Histology atlas of the developing mouse hepatobiliary system with emphasis on embryonic days 9.5-18.5. Toxicol. Pathol., 38, 872–906. 20805319 10.1177/0192623310374329 PMC3490618

[33] Sasaki K. and Sonoda Y. (2000) Histometrical and three-dimensional analyses of liver hematopoiesis in the mouse embryo. Arch. Histol. Cytol., 63, 137–146. 10885450 10.1679/aohc.63.137

[34] Takemura T., Yoshida Y., Kiso S., Saji Y., Ezaki H., Hamano M., Kizu T., Egawa M., Chatani N., Furuta K. et al. (2013) Conditional knockout of heparin-binding epidermal growth factor-like growth factor in the liver accelerates carbon tetrachloride-induced liver injury in mice. Hepatol. Res., 43, 384–393. 22882498 10.1111/j.1872-034X.2012.01074.x

[35] Kobak A.C., Oder G., Kobak Ş.¸., Argin M. and Inal V. (2005) Ochronotic arthropathy: disappearance of alkaptonuria after liver transplantation for hepatitis B-related cirrhosis. J. Clin. Rheumatol., 11, 323–325. 16371803 10.1097/01.rhu.0000191157.25894.55

[36] Introne W.J., Phornphutkul C., Bernardini I., McLaughlin K., Fitzpatrick D. and Gahl W.A. (2002) Exacerbation of the ochronosis of alkaptonuria due to renal insufficiency and improvement after renal transplantation. Mol. Genet. Metab., 77, 136–142. 12359141 10.1016/s1096-7192(02)00121-x

[37] Krechowec S.O., Burton K.L., Newlaczyl A.U., Nunn N., Vlatković N. and Plagge A. (2012) Postnatal changes in the expression pattern of the imprinted signalling protein XLαs underlie the changing phenotype of deficient mice. PLoS One, 7, e29753. 22253771 10.1371/journal.pone.0029753 PMC3256176

[38] Hughes A.T., Milan A.M., Christensen P., Ross G., Davison A.S., Gallagher J.A., Dutton J.J. and Ranganath L.R. (2014) Urine homogentisic acid and tyrosine: simultaneous analysis by liquid chromatography tandem mass spectrometry. J. Chromatogr. B, 963, 106–112. 10.1016/j.jchromb.2014.06.002 24952314

[39] Hughes A.T., Milan A.M., Davison A.S., Christensen P., Ross G., Gallagher J.A., Dutton J.J. and Ranganath L.R. (2015) Serum markers in alkaptonuria: simultaneous analysis of homogentisic acid, tyrosine and nitisinone by liquid chromatography tandem mass spectrometry. Ann. Clin. Biochem., 52, 597–605. 25628464 10.1177/0004563215571969

[40] Frost S.L., Liu K., Li I.M.H., Poulet B., Comerford E., De Val S. and Bou-Gharios G. (2018) Multiple enhancer regions govern the transcription of CCN2 during embryonic development. J. Cell Commun. Signal., 12, 231–243. 29256171 10.1007/s12079-017-0440-4 PMC5842200

[41] Taylor A.M., Preston A.J., Paulk N.K., Sutherland H., Keenan C.M., Wilson P.J.M., Wlodarski B., Grompe M., Ranganath L.R., Gallagher J.A. et al. (2012) Ochronosis in a murine model of alkaptonuria is synonymous to that in the human condition. Osteoarthr. Cartil., 20, 880–886. 22542924 10.1016/j.joca.2012.04.013 PMC3406176

[42] Kranz A., Fu J., Duerschke K., Weidlich S., Naumann R., Stewart A.F. and Anastassiadis K. (2010) An improved Flp deleter mouse in C57Bl/6 based on Flpo recombinase. Genesis, 48, 512–520. 20506501 10.1002/dvg.20641

[43] Moriya K., Bae E., Honda K., Sakai K., Sakaguchi T., Tsujimoto I., Kamisoyama H., Keene D.R., Sasaki T. and Sakai T. (2011) A fibronectin-independent mechanism of collagen fibrillogenesis in adult liver remodeling. Gastroenterology, 140, 1653–1663. 21320502 10.1053/j.gastro.2011.02.005 PMC3081910

[44] Norman B.P., Davison A.S., Ross G.A., Milan A.M., Hughes A.T., Sutherland H., Jarvis J.C., Roberts N.B., Gallagher J.A. and Ranganath L.R. (2019) A comprehensive LC-QTOF-MS metabolic phenotyping strategy: application to alkaptonuria. Clin. Chem., 65, 530–539. 30782595 10.1373/clinchem.2018.295345

